# Intramedullary neurocytomas in the craniocervical spinal cord: A report of two cases and a literature review

**DOI:** 10.3892/ol.2014.2616

**Published:** 2014-10-15

**Authors:** ZHENXING SUN, DAN YUAN, ZHIQIANG CUI, YAXING SUN, JUNSHENG YANG, PENGXIANG YAN, HUANCONG ZUO

**Affiliations:** 1 Department of Neurosurgery, Yuquan Hospital, Medical Center, Tsinghua University, Beijing, P.R. China; 2 Department of Nephrology, The Luhe Teaching Hospital of The Capital Medical University, Beijing, P.R. China; 3 Department of Psychiatry, The Second Municipal Hospital of Zaozhuang City, Zaozhuang, Shandong, P.R. China; 4 Department of Oncology, The Municipal Hospital of Zaozhuang City, Zaozhuang, Shandong, P.R. China; 5 Department of Neurosurgery, Tiantan Hospital, Capital Medical University, Beijing, P.R. China

**Keywords:** central neurocytoma, extraventricular, craniocervical, literature review

## Abstract

Central neurocytoma is a relatively rare tumor of the central nervous system. Young adults are most commonly affected, with a similar incidence in males and females. The tumor is predominantly occurs in the ventricular system of the brain. The tumor is benign and exhibits a good response to surgical resection and radiation therapy. The typical central neurocytoma occurs in the supratentorial ventricular system in young adults. Extraventricular neurocytomas are rare in the spinal cord. In the present study, two cases of craniocervical neurocytomas and the clinical presentation, magnetic resonance imaging observations, pathological features and two-year follow-up results are reported. The first case presents a 26 year old male with an intramedullary mass extending from the medualla oblongata to the T4 segement of the spine. The second case presents the case of a 48 year old female with an intramedullary mass extending from the oblongata to the T2 segement of the spine. The two patients underwent subtotal resection of the masses and post-operative radiotherapy was administered for three months. Post-operative magentic resoance imaging revealed no tumor recurrence in the two cases, two years after resection. The relevant literature is also discussed.

## Introduction

Central neurocytoma is a rare grade II tumor of neuronal origin, according to the World Health Organization staging system (1). Young adults are most commonly affected, with a similar incidence in males and females. The tumors usually occur in the ventricular system of the brain (2). Extraventricular neurocytomas are extremely rare. Since the first case was reported by Hassoun *et al* in 1982 (3), 271 studies regarding ventricular neurocytomas have been published, while only 64 studies regarding extraventricular neurocytoma have been published. By July 2012, extraventricular neurocytoma had been reported in the cerebrum, including the frontal (4), temporal (5,6), parietal (7) and occipital (7) lobes, the pons (8), the skull base (9,10), the vermis of the cerebellum (11), the cerebellum (12), the sellar region (13), the cauda equina (14), the thalamus (15) and the spinal cord (16–25). To date, 17 cases of neurocytoma involving the spinal cord have been reported; nine cases located in the cervical spinal cord (16,17,19,21–23,25) and eight cases located in the thoracic spinal cord (18,20,22,23). The majority of spinal cord neurocytomas do not recur following complete resection and radiation therapy. In the current study, two cases of intramedullary neurocytomas in the craniocervical spinal cord are reported and the clinical features, radiological observations, histopathological presentation and two-year follow-up results are presented, together with a review of the literature. Written informed consent was obtained from both patients.

## Case report

### Case one

A 26-year-old male presented to Yuquan Hospital, Tsinghua University (Beijing, China) with a nine-month history of numbness in the right lower limb and a five-month history of progressive weakness of the left upper limb. The patient’s general health was good and no relevant family history was reported. Upon neurological examination, muscle power in the left upper limb was rated as grade 4/5, according to the Medical Research Council scale (26), with decreased pinprick sensation in the sole of the right foot. Myoatrophy was identified in the right lower limb. A physical examination of the spine did not reveal any abnormalities and perineal sensation was normal.

**Figure 1:**
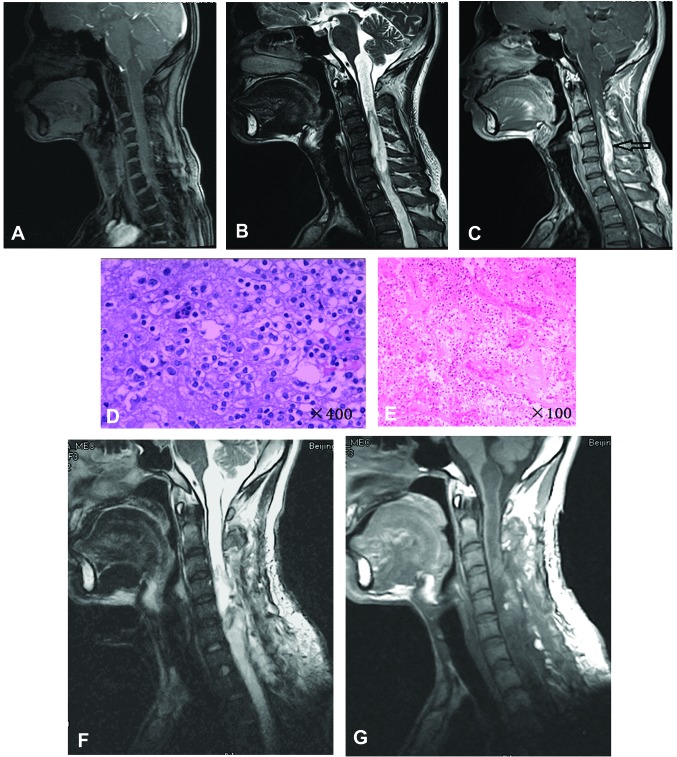
Case one: MRI showing an expansive intramedullary mass extending from the medulla oblongata to the T4 segment of the spine. (A) The mass was isointense on T1-weighted images and (B) hyperintense with a partially cystic mass on T2-weighted images. (C) The mass showed intense heterogeneous enhancement of a solid tumor from the C3 to C7 segments following the injection of gadolinium diethylenetriamine pentaacetic acid. (D and E) Hematoxylin and eosin staining from the biopsy of the mass showed a neoplasm composed of uniform, round cells with round, central nuclei and a perinuclear halo [(D), magnification, ×400; (E), magnification, ×100]. (G and F) Post-operative MRI showing no recurrence of the solid tumor. MRI, magnetic resonance imaging.

A magnetic resonance imaging (MRI) scan of the craniocervical region revealed an expansile intramedullary mass extending from the medulla oblongata to the T4 segment of the spine. The mass was isointense on T1-weighted images, hyperintense with partially cystic mass on T2-weighted images and showed intense heterogeneous enhancement of solid tumor following the injection of gadolinium diethylenetriamine pentaacetic acid (Gd-DTPA) (Fig. 1A–C).

**Figure 2:**
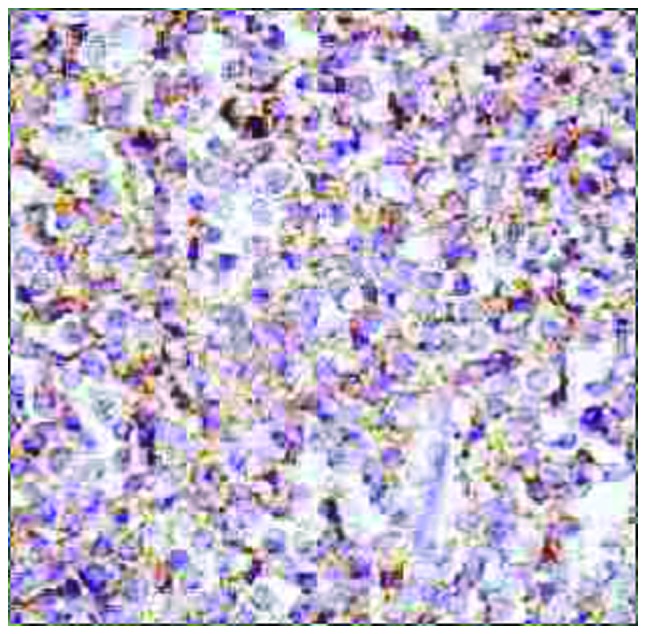
Case one: Immunohistochemical staining revealing positivity for synaptophysin. Magnification, ×400.

A C3-7 laminectomy was performed and an intramedullary solid mass extending from C3 to C7 was exposed. The tumor was gray-purple and exhibited features of infiltrative growth. A partial tumor resection was performed using a micro-neurosurgery technique, resulting in 85% of the tumor being resected. Hematoxylin and eosin staining revealed a neoplasm composed of uniform, round cells (Fig. 1D and E). Immunohistochemical staining revealed positivity for glial fibrillary acidic protein (GFAP), neuronal nuclear antigen, vimentin, neuron-specific enolase (NSE), S-100 protein, synaptophysin (SYN; Fig. 2) and oligo2. Post-operative radiotherapy (56 Gy) was administered for three months. Two years after surgery, the patient’s symptoms were in remission and post-operative MRI revealed no tumor recurrence (Fig. 1F and G).

### Case two

**Figure 3:**
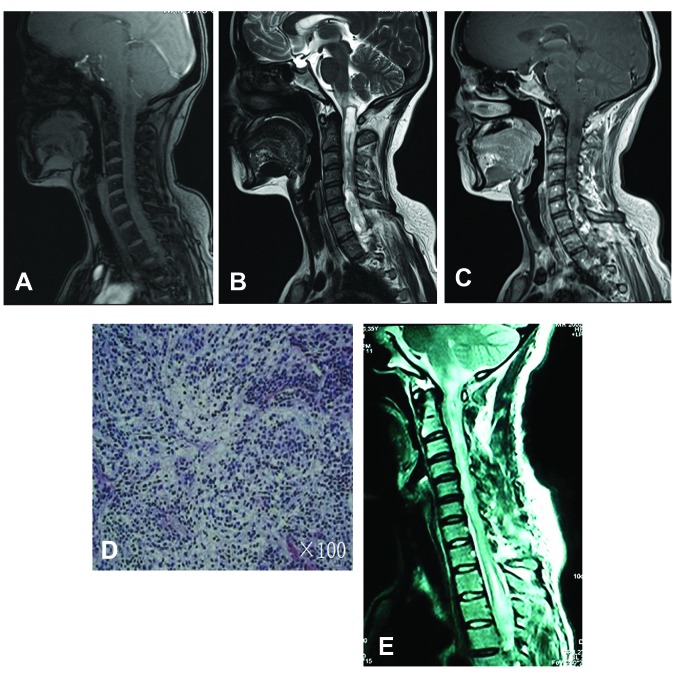
Case two: MRI showing an expansive intramedullary mass extending from the medulla oblongata to the T2 segment. (A) The mass was isointense on T1-weighted images and (B) hyperintense with a partially cystic mass on T2-weighted images. (C) The mass at the C5-T1 level showed intense heterogeneous enhancement of a solid tumor following the injection of gadolinium diethylenetriamine pentaacetic acid. (D) Hematoxylin and eosin staining showed a lesion composed of uniformly small, round cells (magnification, ×100). (E) Post-operative MRI showing residual tumor. MRI, magnetic resonance imaging.

**Figure 4:**
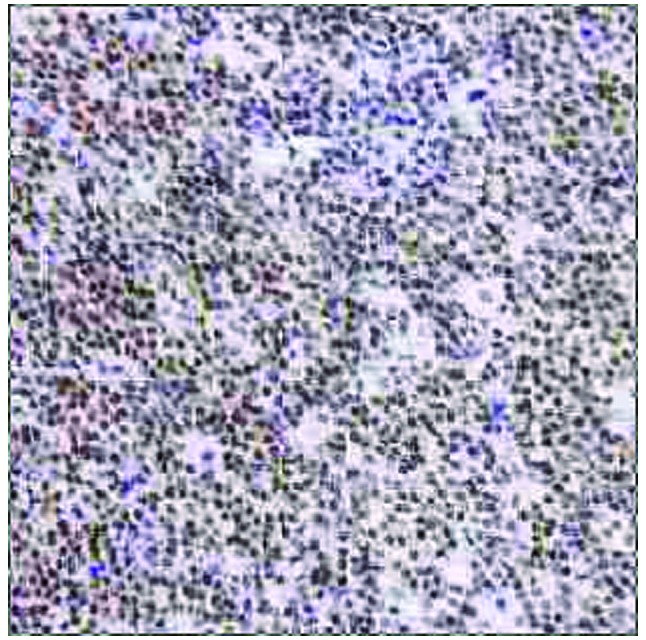
Case two: Immunohistochemical staining revealing positivity for neuronal nuclear antigen. Magnification, ×100.

A 48-year-old female presented to Yuaquan Hospital, Tsinghua University, with a seven-year history of pain in the right lower limb and a five-year history of progressive numbness in all four limbs and lumbar zone anesthesia. MRI revealed a mass extending from the medulla oblongata to the T2 level, with an isointense T1-weighted signal and a hyperintense T2-weighted signal with a partially cystic mass. Intense heterogeneous enhancement of a solid tumor was shown following injection of Gd-DTPA (Fig. 3A–C). The tumor capsule could not be clearly observed around the whole of the tumor, so a subtotal resection of the mass was performed. Hematoxylin and eosin staining revealed a lesion composed of uniformly small, round cells (Fig. 3D). Immunohistochemical staining revealed positivity for GFAP, NSE, vimentin and SYN (Fig. 4). Post-operative radiotherapy (56 Gy) was administered for three months and no deterioration was identified. Following radiotherapy, residual tumor was observed by MRI, however, the patient’s condition had not deteriorated on a follow-up MRI performed after two years (Fig. 3E).

## Discussion

Neurocytomas are tumors of the central nervous system that are derived from the neuronal precursor cells surrounding the central canal region in the developing fetus (2). The spinal cord is an extremely rare site for extraventricular neurocytomas. To date, the majority of cases of spinal neurocytomas cited in the literature have occurred in the cervico-thoracic region. Neurocytomas occurring in the cervical region of the spinal cord region were first documented in 1994 (16). Since then, only eight cases have been reported in the cervical spinal cord (Table I). In the present study, two cases of neurocytoma occurring in the craniocervical region were reported. These are the first cases to be reported in China.

According to the literature review, six cases of cervical spinal neurocytomas occurred in young adults while three cases were reported in adults. In the current study, one patient was 24 years old and the other was 48 years old. The clinical presentation of neurocytoma depends on the location and size of the tumor. Upon neurological examination of case one, the patient was found to have abnormal sensation and loss of proximal muscle power in the upper limbs. MRI revealed a mass with low to intermediate signal intensity on T1-weighted images and intermediate to high signal intensity on T2-weighted images. These tumors involved multiple spinal segments and contrast-enhanced MRI revealed either homogenous or heterogeneous enhancement of the tumor mass. The tumors did not exhibit any characteristic manifestations and their pre-operative diagnosis was relatively difficult, which is typical of spinal neurocytoma. The differential diagnosis of spinal neurocytomas include ependymoma and oligodendroglioma (19).

Neurocytomas are benign and slow growing, and the majority of patients with ventricular neurocytomas may be treated effectively by surgery. However, total resection of spinal cord neurocytomas is relatively difficult, particularly in tumors involving the upper cervical spinal cord and in those involving multiple segments. Giant cervical cord neurocytomas may be treated with subtotal resection followed by radiotherapy, however, in the present literature review, only the two cases reported by Tatter *et al* (16) received radiotherapy. By contrast, Stapleton *et al* (17) reported that post-operative radiotherapy should be avoided, and the remaining six cases did not receive radiotherapy. Of these six cases, one patient succumbed to the tumor and one patient presented with recurrence during follow-up (19,21–23,25). In the present study, for each case, the tumors involved the craniocervical region and were difficult to remove intact. As a consequence, partial and subtotal resections were performed in case one and case two, respectively. Following surgery, the two patients received radiotherapy for three months and no tumor recurrence was observed at the end of the two-year follow-up period. Overall, the number of reported cases of upper cervical neurocytoma is so small that it is unclear whether post-operative radiotherapy is beneficial in preventing tumor recurrence. However, the present study indicates that radiotherapy for a period of three months following surgical resection may prove useful in the prevention of tumor recurrence.

In the present study, the cases of two patients with craniocervical neurocytomas were reported, including the clinical presentation, radiological observations and histopathological features. The successful treatment of neurocytomas may be dependent on an early diagnosis and the total surgical resection of the tumors, however, it is unclear whether post-operative radiotherapy is required to prevent tumor recurrence. Although craniocervical neurocytomas are rare and difficult to diagnose, they must be considered as a presurgical differential diagnosis for craniocervical tumors. In addition, a long-term follow-up period is required for craniocervical neurocytomas due to the possibility of local recurrence.

## Figures and Tables

**Table I:** Summary of the literature review of neurocytomas in the cervical spinal cord.

First author, year (ref.)	Age, years	Location	MRI enhancement	Surgery	Radiotherapy	Recurrence (follow-up time)
Tatter *et al*, 1994 (14)	65	C2-C6	Homogenous	Biopsy	Yes	No (10 years)
	49	C3-C4	Homogenous	Total resection	Yes	Yes (30 months)
Stapleton *et al*, 1997 (15)	12	C4-T1	Heterogeneous	Total resection	No	No (24 months)
Ashkan *et al*, 2000 (20)	12	C6-T1	Homogenous	Subtotal resection	No	No (33 months)
Sharma *et al*, 2006 (1)	24	C5-T1	Homogenous	Total resection	No	Yes (10 months)
Gokham, 2008 (20)	49	C3-C5	Homogenous	Subtotal resection	No	Unknown
Polli *et al*, 2009 (21)	15	C1-T11	Heterogeneous	Subtotal resection	No	Succumbed
	6	C1-C7	Heterogeneous	Subtotal resection	No	No (12 months)
Gepp Rde *et al,* 2012 (23)	15	Cervical spinal cord	Unknown	Subtotal resection	No	Unknown

MRI, magnetic resonance imaging.
